# How Ho Chi Minh City adapted its care pathway to manage the first large-scale community transmission of COVID-19

**DOI:** 10.5365/wpsar.2023.14.5.1045

**Published:** 2023-09-30

**Authors:** Luong Ngoc Khue, Nguyen Trong Khoa, Vuong Anh Duong, Do Thi Hong Hien, Satoko Otsu, Phung Kim Quang, Dereje Abera Ayana, Saho Takaya, Howard L Sobel, Vu Quang Hieu

Viet Nam experienced four waves of coronavirus disease (COVID-19) and by 30 September 2022 had recorded a total of 10.3 million cases and 43 057 deaths. (*1*) Nearly all of Viet Nam’s COVID-19 cases (99.9%) were recorded during the fourth wave, which occurred between April 2021 and September 2022. This report describes actions taken in Ho Chi Minh City (HCMC), the largest city in Viet Nam and the epicentre of the fourth wave, to adapt its care pathway to address the largest surge in cases Viet Nam has faced to date.

## COVID-19 care pathway in the first three waves

The first wave of COVID-19 occurred between 22 January and 22 July 2020, with 415 reported cases and no deaths. (*2*) The second wave commenced on 25 July 2020 and lasted until 27 January 2021, and led to 1136 reported cases, 35 deaths and community transmission in 15 provinces and cities. (*2*) This wave included outbreaks in hospitals and deaths among patients with comorbidities. The third wave was shorter (28 January to 26 April 2021) and resulted in more cases (1301 reported cases) but no deaths. (*2*)

During the first three waves, Viet Nam implemented a “Zero-COVID” strategy. This involved extensive monitoring of new cases and contact tracing, the quarantining of exposed persons, strict lockdowns, hospitalization of all COVID-19 cases, and referral of all severe cases to central hospitals for expert management. This strategy enabled Viet Nam to minimize its COVID-19 deaths in the first year of the pandemic. However, the rapid spread of cases during the fourth wave overwhelmed the Zero-COVID strategy and necessitated a major change in the approach to the management of COVID-19 by everyone involved including doctors, nurses, family members and patients.

## COVID-19 care pathway during the fourth wave

The fourth wave began on 27 April 2021 and was dominated by cases caused by the Delta variant. By May, cases had spread to more than 30 provinces and cities. The largest outbreak was in the industrial parks of Bac Ninh and Bac Giang provinces, where the Delta variant spread extremely rapidly and overwhelmed the ability of hospitals to cope with the surge in case numbers.

To provide comprehensive and integrated care for all patients with COVID-19, regardless of symptoms and severity, Bac Giang province introduced a three-level care pathway. Asymptomatic persons and cases with mild symptoms were monitored at “first-level” field hospitals, located at repurposed district health centres and specialized provincial hospitals. Patients with minimal oxygen requirements or with comorbidities that put them at high risk of severe disease were treated at “second-level” facilities – COVID-19 treatment hospitals without intensive care units (ICUs). Designated COVID-19 treatment hospitals that had ICUs – “third-level” facilities – were reserved for patients with severe and critical COVID-19 disease.

**Fig. 1:**
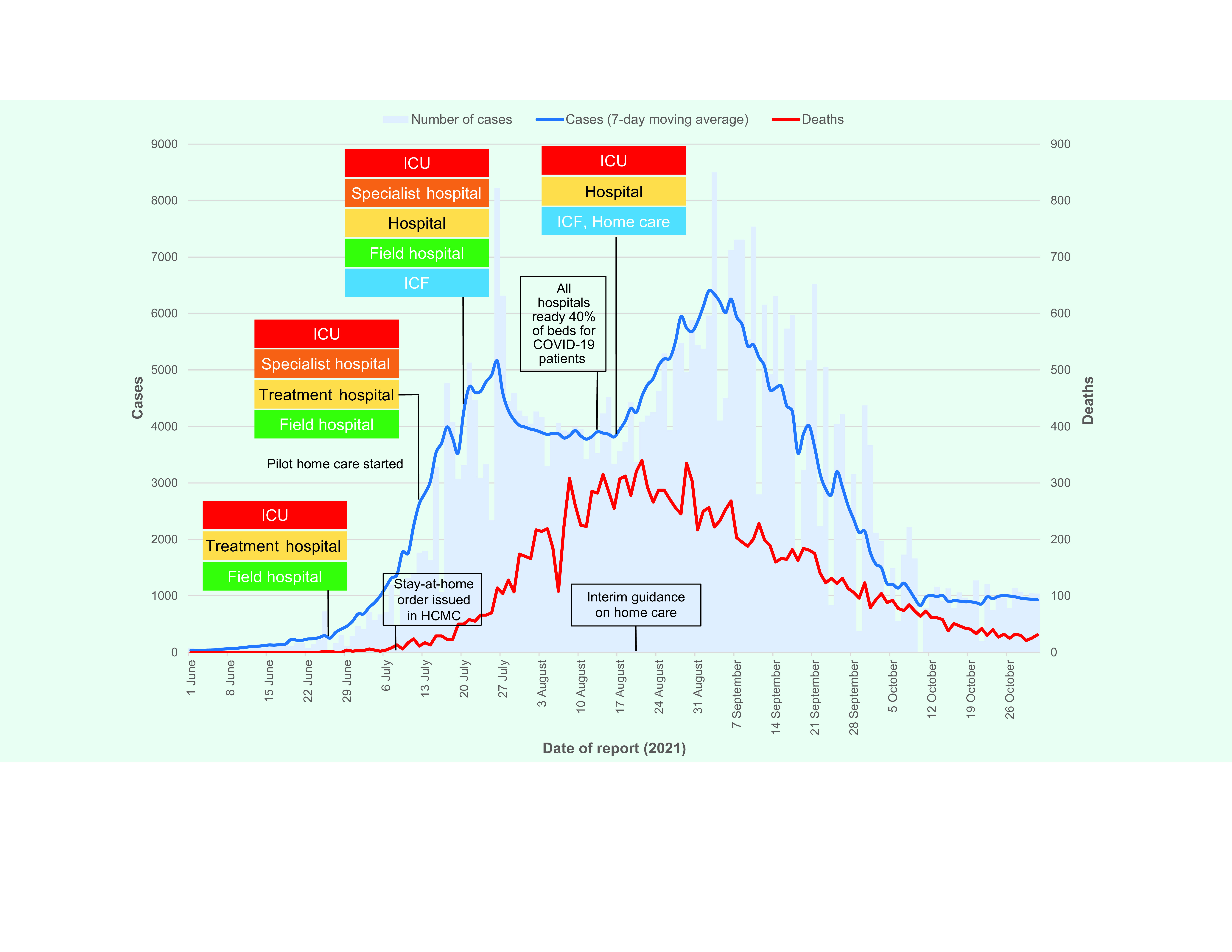
Epidemic curve of COVID-19 cases and deaths with changes made to the care pathway, Ho Chi Minh City, 1 June to 31 October 2021

By June 2021, although Bac Ninh and Bac Giang provinces had managed to bring the outbreak under control, it had already spread to other provinces and cities, including HCMC where it caused the largest surge in Viet Nam. Initially, the city responded by introducing the same three-level care pathway as described above (**Fig. 1**). However, on 9 July 2021, the city issued an absolute stay-at-home order to its citizens (*3*, *4*) and added a fourth level to the care pathway – establishing specialist hospitals for managing COVID-19 patients with severe underlying diseases and who required specialized care. (*5*)

[**insert** Figure 1]

Other parts of the health system were also overwhelmed by the rapid rise in case numbers and required changes. In response to the increase in the volume of emergency calls, including requests for ambulances, HCMC expanded its call centre capacity from 1300 to 5000 calls per day. (*6*) Transportation services were increased from 23 to 323 vehicles by mobilizing 100 ambulances from private businesses and recruiting 200 taxis to take patients newly discharged from hospital back to their homes. (*7*) Taxi drivers were trained in basic infection prevention and control (IPC) measures including hand hygiene, medical mask wearing and car disinfection and cleaning.

On 13 July 2021, HCMC launched a pilot programme to provide home care for those with asymptomatic COVID-19. At the same time, more than 10 000 health-care workers (HCWs) from across the country were called upon to support the response in HCMC. (*8*) The Ministry of Health played a key role in the deployment of HCWs from other provinces, communicating closely with health-care facilities in HCMC to understand their needs and training HCWs in IPC and nursing care for COVID-19 patients before their deployment in HCMC. HCWs were closely monitored for onset of COVID-19 symptoms and underwent twice daily temperature checks.

When the daily average number of new cases surpassed 3000, HCMC reconfigured the care pathway for a second time by introducing a fifth level (**Fig. 1**). (*9*) Hotels, dormitories, schools and other public facilities were used as intermediate care facilities (ICFs) to treat those with asymptomatic infection or mild symptoms without underlying diseases or risk factors. ICFs were equipped to treat mild cases and to stabilize emergency cases before referral to a higher-level facility. Additionally, asymptomatic persons who were homeless, without caregivers or unable to implement IPC measures at home were admitted to ICFs.

On 16 August 2021, as COVID-19 cases continued to increase, HCMC simplified the care pathway, reducing the number of levels from five back to three (**Fig. 1**). As part of the reconfiguration, home care and ICFs were combined to deliver first-level care. Case numbers started decreasing in September 2021. During the 5 months from June to October 2021, HCMC reported a total of 430 209 cases and 16 551 deaths (a case–fatality ratio of 3.8%). (*1*)

## Additional measures during the fourth wave

In addition to the timely reconfigurations of the care pathway described above (**Fig. 1**), the following evidence-based measures and support systems were introduced in HCMC to address the surge in COVID-19 cases during the fourth wave and minimize the impact on the health system: (*8*, *10*)

The government mobilized 133 000 military personnel and 126 000 police officers.
HCMC established 536 mobile health stations to provide rapid tests, vaccinations, first aid and referrals.
A “network of physician companions” consisting of more than 10 000 medical staff and volunteers across the country provided counselling, health education and psychological support to COVID-19 patients and their families.
By providing staff and equipment, central hospitals across the country helped to establish 12 ICU centres in HCMC, creating 7900 beds including 3000 intensive care beds.
A warehouse was set up to supply medicines, equipment and other medical consumables to localities across HCMC.
An online dashboard for real-time bed occupancy was established and made available to the public by the HCMC health department. This allowed the public to make more informed decisions about which hospital to go to for treatment.
All hospitals, both public and private, were instructed to make up to 40% of their beds available for COVID-19 patients during the fourth wave.
Interim guidance was issued on home care and the organization of mobile health stations, including the proper use of medication in home care.
Political party authorities and other political leaders enhanced community engagement on COVID-19 response measures.

Furthermore, government authorities issued updated guidance to support adaptations to the care pathway in response to the evolving pandemic. (*11*) This information was widely disseminated in a timely manner through regular and ad hoc meetings and via the city’s web site. Encouragement of community engagement by local leaders contributed to better understanding of COVID-19 and preventive measures. (*11*)

## Discussion

In August 2021, at the peak of the fourth wave, HCMC recorded almost 8500 cases of COVID-19 in one day. Throughout the outbreak, both HCMC and the Viet Nam Ministry of Health demonstrated a strong commitment to combating COVID-19 and acted in a timely manner to address the increased demand for COVID-19 case management and referrals using a multisectoral approach. In HCMC, the COVID-19 care pathway was reconfigured several times, while thousands of HCWs and volunteers were mobilized to staff new mobile health units, the expanded ICU capacity and the many thousands of COVID-19 beds that were created as part of the COVID-19 response.

During the fourth wave of the pandemic, HCMC shifted away from a centralized, hospital-based case management model towards an integrated care pathway that used multiple levels of health care, including home care and ICFs. Based on the overall framework issued by the Ministry of Health, HCMC continually adapted its care pathway to respond to the real-time ongoing situation. This flexible approach to the care pathway ensured that the health-care delivery system was able to treat the “right patient at the right time,” while avoiding being overwhelmed. High-level commitment and leadership ensured that both the government and society did their part to respond to the pandemic. This was key to HCMC’s successful response to the fourth wave of COVID-19. (*12*)

In conclusion, the ability to make timely adjustments to the care pathway in response to rapidly changing local contexts through multisectoral engagement and high-level commitment helped Viet Nam to mount a successful COVID-19 pandemic response without overwhelming the health-care system.
